# Supplementary material for: Hydrogen Sulfide Treatment Improves Post-Infarct Remodeling and Long-Term Cardiac Function in CSE Knockout and Wild-Type Mice
Source: Int J Mol Sci. 2020 Jun 16;21(12):4284. doi: 10.3390/ijms21124284 (PMC7352717; doi:10.3390/ijms21124284)
Supplement: Supplementary file 1 [file ijms-21-04284-s001.pdf]

SUPPLEMENTARY MATERIAL

Hydrogen Sulphide Treatment Ameliorates Post-Infarct Remodelling and Improves Long-Term Cardiac Function in Cystathionine γ-lyase Knockout and Wild-type Mice

Leigh J. Ellmers, PhD [1], Evelyn M Templeton, MClinIm [1], Anna P Pilbrow, PhD [1], Chris Frampton, PhD [1], Isao Ishii, PhD [2], Philip K Moore, PhD [3], Madhav Bhatia, PhD [4], A. Mark Richards, MD, PhD [1, 5], and Vicky A. Cameron, PhD [1 *]

Corresponding Author: Professor Vicky Cameron, Department of Medicine, University of Otago, Christchurch, 2 Riccarton Avenue, Christchurch, P.O. Box 4345, Christchurch 8140, New Zealand Email: Vicky.cameron@otago.ac.nz

Supplementary Table 1: Expression of Atrial mir-21 and Ventricular Genes Relative to WT Sham Vehicle Group (Medians (Interquartile Ranges))

Gene	WT Sham Vehicle	WT Sham GYY4137	WT Infarct Vehicle	WT Infarct GYY4137	CSE$^{-/-}$ Sham Vehicle	CSE$^{-/-}$ Sham GYY4137	CSE$^{-/-}$ Infarct Vehicle	CSE$^{-/-}$ Infarct GYY4137
**Atrial gene expression**								
**miR-21**	1.00 (0.72, 2.05)	1.06 (0.61, 1.83)	2.95 (1.72, 3.63) †	1.96 (1.38, 3.76)	2.77 (2.63, 3.54) ‡‡	3.88 (1.88, 7.15)	4.35 (2.12, 6.93) †	4.83 (2.20, 6.61)
**Ventricular gene expression**								
**ANP** (*Nppa*)	1.00 (0.78, 2.65)	0.81 (0.67, 1.34)	3.35 (3.32, 3.42) †	1.51 (1.31, 1.54) *	1.94 (0.83, 5.67)	1.24 (0.86, 2.60)	3.24 (1.79, 4.43) †	0.89 (0.41, 1.26) *
**BNP** (*Nppb*)	1.00 (0.86, 1.17)	1.35 (0.67, 1.66)	2.03 (1.71, 2.23)	0.64 (0.53), 0.95) *	0.87 (0.53, 1.16)	1.19 (0.82, 1.55)	1.90 (1.13, -)	0.72 (0.31, 1.40)
**βMHC** (*Myh7*)	1.00 (0.41, 1.36)	0.97 (0.65, 3.44)	3.54 (3.13, 3.75) †	0.42 (0.35, 1.43) **	0.92 (0.55, 1.13)	1.04 (0.59, 1.81)	1.89 (1.18, 4.44) †	0.73 (0.58, 1.10) *
**αMHC** (*Myh6*)	1.00 (0.63, 1.17)	1.27 (1.24, 1.65)	1.73 (1.50, 2.02) †	0.72 (0.60, 1.22) *	0.89 (0.46, 1.22)	1.37 (0.94, 1.69)	1.39 (1.05, 1.79)	0.74 (0.65, 0.95) *
**TGFβ1 (***Tgfb1)*	1.00 (0.77, 1.09)	0.92 (0.65, 1.66)	2.91 (2.16, 3.02) ††	0.71 (0.54, 1.63) **	0.90 (0.78, 0.97)	1.04 (0.76, 1.25)	1.99 (1.67, 2.99) ††	0.50 (0.35, 0.96) **
**GATA4 (***Gata4)*	1.00 (0.58, 1.14)	0.90 (0.84, 1.58)	1.60 (1.43, 1.91)	0.46 (0.37, 1.67) *	0.84 (0.57, 0.98)	1.33 (0.71, 1.48)	1.21 (0.94, 1.71)	0.52 (0.32, 0.81) *
**Collagen 1a1 (***Col1a1)*	1.00 (0.63, 1.18)	1.55 (0.86, 2.50)	4.34 (3.43, 5.23) †	1.01 (0.75, 2.67) *	0.92 (0.63, 0.96)	1.56 (0.92, 5.96)	2.83 (1.74, 4.35) †	0.86 (0.50, 1.53) *
**NRF (***Nrf2)*	1.00 (0.69, 1.12),	1.37 (1.29, 2.47)	--	1.70 (1.20, 2.08)	1.07 (0.60, 1.11)	1.24 (1.08, 1.76)	1.25 (1.07, 1.58)	0.97 (0.65, 1.50)
**AT1R** (*Agtr1*)	1.00 (0.91, 1.45)	1.47 (1.14, 1.74)	1.91 (1.83, 2.06) †	0.63 (0.49, 2.37) *	1.11 (0.76, 1.27)	1.64 (1.48, 1.94)	1.64 (1.38, 2.47) †	0.67 (0.33, 1.64) *
**ACE** (*Ace*)	1.00 (0.71, 1.32)	1.29 (1.08, 2.24)	5.00 (4.01, 6.01) ††	1.16 (0.93, 1.72) **	0.89 (0.69, 1.19) ‡	1.35 (1.18, 2.31)	1.65 (1.25, 2.90) ‡‡	0.63 (0.39, 1.20) *
**AGT** (*Agt*)	1.00 (0.77, 1.35)	1.35 (1.07, 1.41)	0.89 (0.64, 1.00)	0.69 (0.54, 2.08)	1.14 (0.95, 1.36)	1.41 (1.01, 3.40)	1.65 (1.19, 3.23)	0.73 (0.48, 1.14)
**Caspase 3** (*Casp3*)	1.00 (0.71, 1.25)	1.19 (1.14, 1.72)	3.00 (2.31, 3.45) ††	1.15 (1.03, 1.75) *	0.91 (0.62, 1.11)	1.27 (1.22, 2.28)	1.37 (1.01, 2.10) ‡‡	0.65 (0.28, 1.06) *
**Akt1** (*Akt1*)	1.00 (0.72, 1.36)	1.58 (1.34, 2.25)	1.96 (1.87, 2.05) †	0.99 (0.95, 1.80)	0.97 (0.63, 1.08)	1.54 (1.41, 2.10)	1.60 (1.00, 2.59) †	0.75 (0.48, 1.10) *

* $p<0.05$, ** $p<0.001$ for GYY4137 vs vehicle; † $p<0.05$, †† $p<0.001$ for effect of sham vs infarct; ‡ $p<0.05$, ‡‡ $p<0.001$ for wild type vs CSE$^{-/-}$

Supplementary Table 2: Expression of Kidney Genes Relative to WT Sham Vehicle Group (Medians (Interquartile Ranges))

Gene	WT Sham Vehicle	WT Sham GYY4137	WT Infarct Vehicle	WT Infarct GYY4137	$CSE^{-/-}$ Sham Vehicle	$CSE^{-/-}$ Sham GYY4137	$CSE^{-/-}$ Infarct Vehicle	$CSE^{-/-}$ Infarct GYY4137
**ANP** (*Nppa*)	1.00 (0.86, 1.11)	0.47 (0.39, 0.75) *	1.62 (1.18, 1.82) †	1.13 (0.91, 1.37)	0.70 (0.59, 1.05)	0.97 (0.71, 1.12)	1.00 (0.83, 1.41) ‡	0.77 (0.70, 1.08)
**TGFβ1 (***Tgfb1)*	1.00 (0.88, 1.06)	0.38 (0.31, 0.56)	1.69 (1.38, 3.05) †	0.76 (0.56, 0.94) *	0.77 (0.60, 1.10)	0.75 (0.48, 1.30)	0.80 (0.66, 1.17)	0.82 (0.62, 1.48)
**Collagen1a1 (***Col1a1)*	1.00 (0.75, 1.17)	0.51 (0.46, 0.67)	2.07 (1.78, 2.76) †	0.55 (0.37, 0.76) **	0.59 (0.46, 0.89)	0.74 (0.45, 0.89)	0.98 (0.62, 1.05)	0.73 (0.40, 1.05)
**AT1R** (*Agtr1*)	1.00 (0.69, 1.23)	0.50 (0.42, 0.63) *	1.12 (1.05, 1.22)	0.94 (0.71, 0.96)	0.92 (0.65, 1.01)	0.86 (0.48, 0.98)	0.89 (0.62, 1.04)	0.79 (0.49, 0.98)
**AGT** (*Agt*)	1.00 (0.84, 1.18)	0.72 (0.49, 0.86)	1.07 (0.94, 1.14)	0.77 (0.72, 0.97)	1.01 (0.75, 1.51)	1.42 (1.01, 1.61) ‡	1.15 (0.90, 1.57) ‡	1.20 (1.01, 2.47)
**ACE** (*Ace*)	1.00 (0.83, 1.29)	0.41 (0.38, 0.45) *	1.34 (1.23, 1.39)	0.95 (0.58, 1.03) *	0.73 (0.67, 1.55)	0.80 (0.70, 1.20)	0.92 (0.65, 1.80)	0.56 (0.38, 0.79)
**Caspase 3** (*Casp3*)	1.00 (0.74, 1.18)	0.52 (0.50, 0.72) †	0.52 (0.50, 0.55)	0.45 (0.39, 0.60)	0.86 (0.63, 1.32)	0.79 (0.72, 1.24)	1.04 (0.75, 1.28) ‡	1.06 (0.81, 1.27) ‡
**Akt1** (*Akt1*)	1.00 (0.68, 1.21)	0.52 (0.48, 0.72)	0.80 (0.61, 0.96)	0.66 (0.58, 0.79)	0.72 (0.49, 1.25)	0.87 (0.80, 1.18)	1.08 (0.68, 1.19)	1.00 (0.70, 1.36)

* $p<0.05$, ** $p<0.001$ for GYY4137 vs vehicle; † $p<0.05$, †† $p<0.001$ for effect of sham vs infarct; ‡ $p<0.05$, ‡‡ $p<0.001$ for wild type vs $CSE^{-/-}$

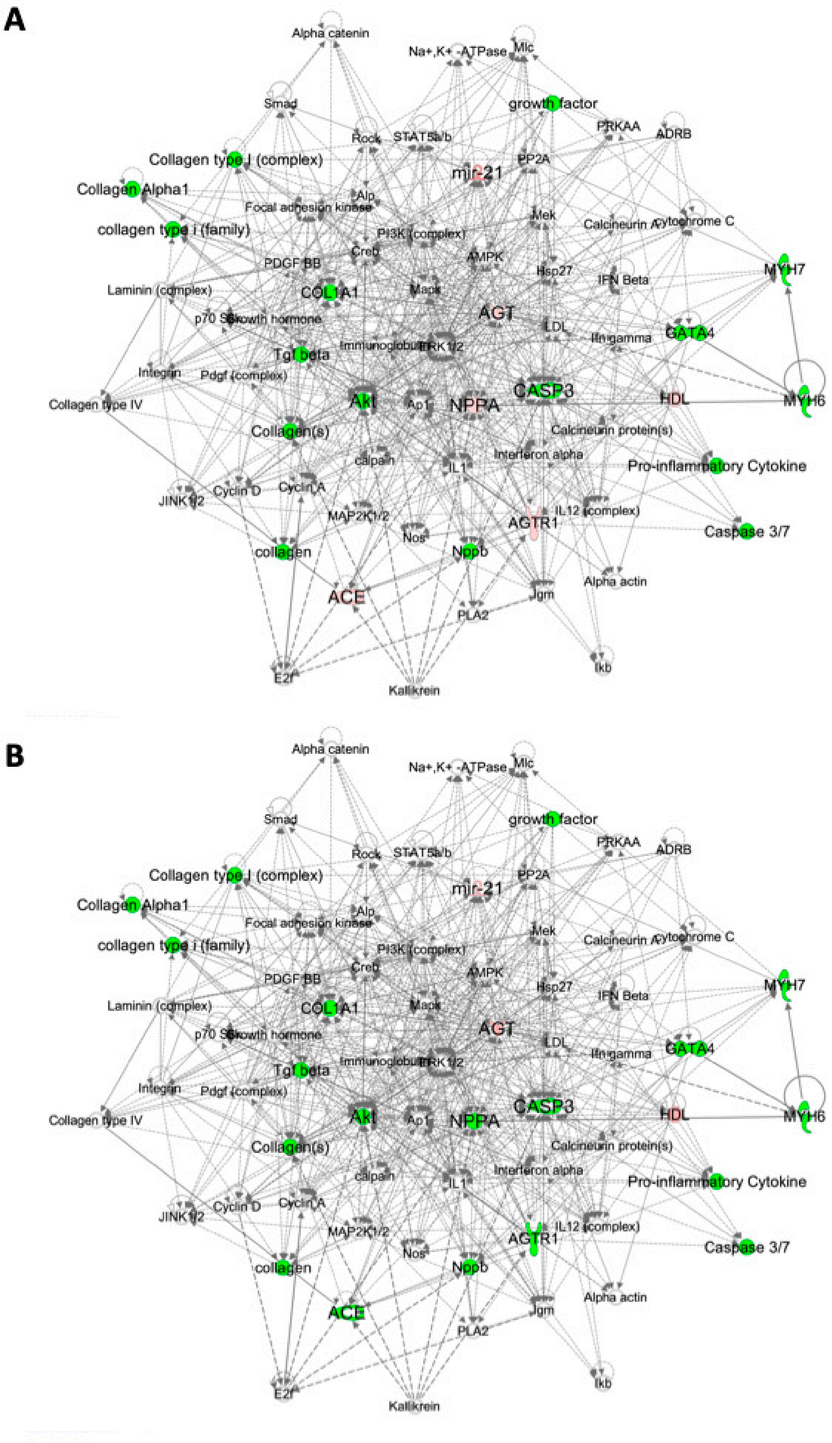

Supplementary Fig 1: Gene networks among cardiac genes comparing wildtype with CSE$^{-/-}$ mice

Gene networks illustrating direct and indirect functional relationship between cardiac genes comparing WT with CSE$^{-/-}$mice: (A) WT Sham Vehicle vs CSE$^{-/-}$ Sham Vehicle; (B) WT Infarct Vehicle vs CSE$^{-/-}$ Infarct Vehicle. Highlighted genes have higher (red) or lower (green) expression in the second treatment group compared to the first group, for each pair. The colour intensity correlates with the magnitude of the fold-change in the expression between treatment groups. Highlighting does not necessarily indicate a statistically significant fold-change in expression between the groups. Gene networks were generated through the use of IPA (QIAGEN Inc., https://www.qiagenbio- informatics.com/products/ingenuity-pathway-analysis).

Supplementary Table 3: Real-time PCR primer sequences and annealing temperatures

Gene Name<br>**(*Gene Symbol)***	Primers	Temperature
Collagen 1 (*Col1a1*)	f AGGCTTCAGTGGTTTGGATG<br>r CACCAACAGCACCATCGTTA	54 °C
Transforming growth factor β1 (*Tgfb1*)	f TGAGTGGCTGTCTTTTGACG<br>r GGTTCATGTCATGGATGGTG	54 °C
β-Myosin heavy chain (*Myh7*)	f TTGGATGAGCGACTCAAAAA<br>r GCTCCTTGAGCTTCTTCTGC	60 °C
α-Myosin heavy chain (*Myh6*)	f TGAGCTGGAAGAAAAGCTC<br>r GGTTTTCCTTCAGTTTCTT	60 °C
Angiotensin-converting enzyme (*Ace*)	f TGCCTCCCAAGGAATTAGAA<br>r CCATGCCCATAGCAATTCTT	59 °C
Angiotensinogen (*Agt*)	f AAGACCTCCCCTGTGAATGA<br>r CCTGCCTCATTCAGCATCTT	65 °C
Angiotensin type 1 receptor (*Agtr1*)	f GGAAACAGCTTGGTGGTGAT<br>r ACATAGGTGATTGCCGAAGG	63 °C
ANP (*Nppa*)	f GAACCTGCTAGACCACCT<br>r CCTAGTCCACTCTGGGCT	56 °C
BNP (*Nppb*)	f AAGCTGCTGGAGCTGATAAGA<br>r GTTACAGCCCAAACGACTGAC	56 °C
Thymoma viral proto-oncogene 1 (*Akt1*)	f CATGAACGACGTAGCCATTG<br>r AAGGTGCCATCGTTCTTGAG	51 °C
GATA-4 (*Gata4*)	f AATGCCTGTGGCCTCTATCA<br>r CTGGTTTGAATCCCCTCCTT	58 °C
Nuclear Factor, Erythroid 2 Like 2 (*Nrf2*)	f GTCAAACAGAACGGCCCTAA<br>r CACATTGGGATTCACGCATA	50 °C
Caspase 3 (*Casp3*)	f CGGCGTGTTTCTGTTTTGTT<br>r TGCATTGCTAGGCAGTCGTA	51 °C

Sequences are listed 5′ to 3′. Forward primers are designated by f and reverse primers by r.

Supplementary Table 4: Correlations of raw normalised versus ventricular expression data

Gene	Raw samples, N	Normalised Samples, N	Pearson Correlation	p-value
Nppa	53	41	0.868 **	<0.0001
Nppb	49	39	0.861 **	<0.0001
Myh7	51	39	0.890 **	<0.0001
Myh6	53	41	0.882 **	<0.0001
Tgfb1	53	37	0.750 **	<0.0001
Gata4	53	41	0.887 **	<0.0001
Col1a1	53	41	0.932 **	<0.0001
Nrf2	39	39	0.737 **	<0.0001
Agtr1	53	41	0.824 **	<0.0001
Ace	53	41	0.893 **	<0.0001
Agt	53	41	0.885 **	<0.0001
Casp3	51	39	0.888 **	<0.0001
Akt1	51	39	0.902 **	<0.0001

Gene expression data were skewed and natural log transformed for Pearson correlation analysis
** Pearson Correlation is statistically significant at $p < 0.0001$.
